# Simple Electrospinning Method for Biocompatible Polycaprolactone β-Carotene Scaffolds: Advantages and Limitations

**DOI:** 10.3390/polym16101371

**Published:** 2024-05-11

**Authors:** Orion Yoshikawa, Valentina Basoli, Francesco Boschetto, Alfredo Rondinella, Alex Lanzutti, Wenliang Zhu, Enrico Greco, Florian Markus Thieringer, Huaizhong Xu, Elia Marin

**Affiliations:** 1 Ceramic Physics Laboratory, Faculty of Materials Science and Engineering, Kyoto Institute of Technology, Sakyo-ku, Matsugasaki, Kyoto 606-8585, Japan; m2672032@edu.kit.ac.jp (O.Y.); wlzhu@kit.ac.jp (W.Z.); 2 Medical Additive Manufacturing Research Group (Swiss MAM), Department of Biomedical Engineering, University of Basel, Hegenheimermattweg 167C, 4123 Allschwil, Switzerland; valentina.basoli@unibas.ch (V.B.); f.thieringer@unibas.ch (F.M.T.); 3 Center for Excellence in Hip, Scottish Rite for Children, Dallas, TX 75219, USA; boschetto.cesc@gmail.com; 4 Department of Orthopedic Surgery, UT Southwestern Medical Center, Dallas, TX 75390, USA; 5 Polytechnic Department of Engineering and Architecture, University of Udine, 33100 Udine, Italy; alfredo.rondinella@uniud.it (A.R.); alex.lanzutti@uniud.it (A.L.); 6 Department of Chemical and Pharmaceutical Sciences, University of Trieste, 34127 Trieste, Italy; enrico.greco@units.it; 7 National Interuniversity Consortium of Materials Science and Technology (INSTM), Trieste Research Unity, Via G. Giusti 9, 50121 Firenze, Italy; 8 Clinic of Oral and Cranio-Maxillofacial Surgery, University Hospital Basel, 4031 Basel, Switzerland; 9 Department of Biobased Materials Science, Kyoto Institute of Technology, Sakyo-ku, Matsugasaki, Kyoto 606-8585, Japan; xhz2008@kit.ac.jp; 10 Biomaterials Engineering Laboratory, Kyoto Institute of Technology, Sakyo-ku, Matsugasaki, Kyoto 606-8585, Japan; 11 Biomedical Research Center, Kyoto Institute of Technology, Sakyo-ku, Matsugasaki, Kyoto 606-8585, Japan; 12 Materials Innovation Laboratory, Kyoto Institute of Technology, Sakyo-ku, Matsugasaki, Kyoto 606-8585, Japan

**Keywords:** electrospinning, polycaprolactone, β-carotene, scaffolds, tissue engineering, cellular proliferation, antibacterial

## Abstract

In this study, electrospun scaffolds were fabricated using polycaprolactone (PCL) loaded with varying concentrations of β-carotene (1.2%, 2.4%, and 3.6%) via the electrospinning technique. The electrospinning process involved the melting of PCL in acetic acid, followed by the incorporation of β-carotene powder under constant stirring. Raman spectroscopy revealed a homogeneous distribution of β-carotene within the PCL matrix. However, the β-carotene appeared in particulate form, rather than being dissolved and blended with the PCL matrix, a result also confirmed by thermogravimetric analysis. Additionally, X-ray diffraction analysis indicated a decrease in crystallinity with increasing β-carotene concentration. Mechanical testing of the scaffolds demonstrated an increase in ultimate strain, accompanied by a reduction in ultimate stress, indicating a potential plasticizing effect. Moreover, antimicrobial assays revealed a marginal antibacterial effect against *Escherichia coli* for scaffolds with higher β-carotene concentrations. Conversely, preliminary biological assessment using KUSA-A1 mesenchymal cells indicated enhanced cellular proliferation in response to the scaffolds, suggesting the potential biocompatibility and cell-stimulating properties of β-carotene-loaded PCL scaffolds. Overall, this study provides insights into the fabrication and characterization of electrospun PCL scaffolds containing β-carotene, laying the groundwork for further exploration in tissue engineering and regenerative medicine applications.

## 1. Introduction

Tissue engineering holds tremendous promise in regenerative medicine by offering innovative solutions for repairing and replacing damaged tissues and organs [1]. Central for this field is the development of scaffolds [2], which serve as three-dimensional templates to support cell adhesion [3], proliferation [4], and tissue regeneration [5]. Among various scaffold fabrication techniques, electrospinning is notable for its versatility in producing nanofibrous structures that mimic the extracellular matrix (ECM) of natural tissues [6].

Electrospinning, a technique initially developed in the early 20th century, has gained prominence in recent years for its ability to fabricate nanofibrous scaffolds with high surface area-to-volume ratios and controllable fiber diameters [7]. The process involves the use of an electric field to draw a polymer solution or melt from a syringe tip, resulting in the formation of ultrafine fibers as the solvent evaporates or solidifies. Electrospinning offers flexibility in material selection, with various polymers, including synthetic polymers like polycaprolactone (PCL), as well as natural polymers such as collagen and gelatin, being compatible with the process [8].

PCL has emerged as one of the most favored polymers for electrospinning due to its excellent processability, biodegradability, and biocompatibility [9]. However, PCL has limitations such as poor cellular adhesion [10] and a lack of inherent bioactive properties, which may hinder its effectiveness in promoting tissue regeneration. To overcome these drawbacks, researchers have explored surface treatments [10] and the incorporation of bioactive substances into PCL scaffolds to enhance their biological functionality [11,12,13,14].

Carotenoids, a diverse group of naturally occurring pigments found abundantly in fruits [15], vegetables [16], and certain microorganisms [17], possess a remarkable array of bioactive properties that hold potential in various biomedical applications. Carotenoids offer significant protection against free radical damage due to their ability to scavenge reactive oxygen species (ROS) [18], which is crucial for maintaining cellular health and function and potentially aids in preventing chronic diseases like cardiovascular disorders [19], cancer [20], and neurodegenerative conditions [21]. Studies have shown their effectiveness in reducing inflammation associated with various conditions like arthritis [22], asthma [23], and inflammatory bowel disease [24]. Specific carotenoids also exhibit the ability to stimulate cell proliferation and differentiation, essential processes in tissue regeneration and wound healing, making them promising candidates for promoting tissue repair and growth in various medical contexts. Among different carotenoids, β-carotene was chosen due to its well-established biological activities, readily available source, and established safety profile, making it a promising candidate for incorporation into scaffolds for biomedical applications.

β-carotene, a natural antioxidant and precursor of vitamin A, has garnered significant attention for its potential biomedical applications, including antioxidant [25], anti-inflammatory [26], and cell-stimulating properties [27,28]. Incorporating β-carotene into PCL scaffolds represents a promising strategy to imbue them with bioactive characteristics, thereby enhancing their suitability for tissue engineering applications [29,30,31,32].

In this context, the present study aims to fabricate β-carotene-loaded PCL scaffolds using the electrospinning technique and investigate their potential for biomedical applications, particularly for tissue regeneration. This research, based on a very simple material production method, explores the influence of varying β-carotene concentrations on the morphology, physicochemical properties, mechanical characteristics, and biological responses of the scaffolds. Based on the literature on similar composite materials, it is hypothesized that the incorporation of β-carotene into PCL scaffolds could not only improve their biocompatibility and promote cell interaction but also preserve the mechanical integrity of the matrix, rendering them versatile platforms for tissue engineering and regenerative medicine endeavors.

## 2. Materials and Methods

### 2.1. Electrospinning Process

For the production of the base material, 400 mg of PCL (Mw ~80,000, Sigma Aldrich, St. Louis, MO, USA) was added to 2 mL of acetic acid (Nacalai Tesque, Kyoto, Japan) and stirred for 12 h. For the composites, 5 mg, 10 mg, and 15 mg (about 1.2%, 2.4%, and 3.6%, respectively) of β-carotene powder (1 µm average particle diameter, Nacalai Tesque, Kyoto, Japan) were added to the solution and stirred for an additional hour.

Printing parameters were chosen from a previous work [33]: 1 mL of solution was added to a syringe with a 23G needle and injected at a rate of 0.2 μm/s (flow rate: 3.5 × 10^−3^ mm^3^/s) under an applied voltage of 10 kV while keeping the metal needle at a fixed distance of 5 cm from the target aluminum foil cathode. The samples were produced under controlled environmental conditions (T = 25 °C and RH = 25–35%).

### 2.2. Characterization Techniques

#### 2.2.1. Confocal Imaging

Micrographs of the samples’ surfaces were taken using a 3D laser-scanning microscope (VKX200K series, Keyence, Osaka, Japan) with magnifications ranging from 10× to 150× and a numerical aperture between 0.30 and 0.95. Surface maps obtained from the merging of different images could be acquired by using a dedicated automated xy stage combined with the autofocus function for the z axis.

Phase-contrast and fluorescence images of cell viability and morphology were acquired using a Nikon AXRGH confocal microscope (Nikon, Japan) equipped with a CCD camera at magnifications of 10× (0.16) and 20× (water immersion objective 0.8).

#### 2.2.2. Scanning Electron Microscope

An SM-700 1F Scanning Electron Microscopy (JEOL, Tokyo, Japan) device was employed to capture high-magnification images of the samples both pre- and post-biological testing. Prior to observation, the samples underwent sputter-coating with a platinum layer (approximately 2 nm thick) and were subsequently examined at an accelerating voltage of 10 kV.

#### 2.2.3. Spinnability Test

The spinnability of the solutions was evaluated with a simple pull-out (pull-up) test, where a polyethylene cylindrical probe (2 mm diameter) was immersed in the electrospinning solution to a depth of 1 cm and then pulled out from the solution at a speed of 500 mm/min. A fiber will then form between the meniscus of the solution and the tip, and the maximum length of this fiber depends on the cohesive forces between the molecules of the solution and its adhesion to the probe. The length of the fiber at the breaking point can then be used to qualitatively estimate the ability of the solution to form fibers.

The devices used for these assessments (separate from the electrospinning one), together with test parameters, were designed through an optimization process. Polyethylene was chosen as the material for the probe because of its adhesion to the studied solutions, so that the pull-out test could be carried out. The probe’s chosen diameter allows the formation of fibers with a cross-section that can withstand tension without immediate rupture, so the differences between the cohesive forces of different solutions can be appreciated. The depth, on the other hand, allows material to be taken from the core of the solution, in order to have a homogeneous composition. The pull-out speed was chosen to make the influence of gravity on the test results non-significant. 

#### 2.2.4. Raman Spectroscopy

Raman imaging was conducted utilizing a confocal Laser Raman microscope (RAMANtouch, Nanophoton Co., Ltd., Osaka, Japan) with excitation sources at 532 nm and a nominal power of 200 mW. To mitigate the risk of sample burning, the power output was regulated by adjusting a dedicated ND filter. The micro-probe employed lenses ranging from 5× to 100× magnifications, with numerical apertures spanning from 0.5 to 0.23. 

Imaging involved the acquisition of linear arrays (x axis) consisting of 400 points, which were subsequently combined into a bi-dimensional map (y axis). 

The average spectra for each material were then analyzed and deconvoluted using dedicated software (Labspec 5.0, Horiba, Kyoto, Japan).

#### 2.2.5. X-ray Diffraction

XRD analyses were performed on a Rigaku Ultima IV (Rigaku Corporation, Tokyo, Japan), using CuKa radiation. Diffraction patterns were acquired in the range of 5°–50° with a step size of 0.02 at a rate of 3°/min. The penetration depth of the XRD probe was in the order of 1 mm. For PCL, the peaks at about 22° [110] and 24° [200] [34] were considered representative of the crystalline phase, while the main peaks related to β-carotene could be found at about 24°, 24.5°, 25.5°, 28.5°, and 29.5° [35]. The crystallinity index was calculated as the ratio between the integrated intensity of the crystalline peaks and the total integrated intensity after subtraction of the β-carotene contribution, using the following equation:

$$X_{c}=\frac{I_{PCL}}{I_{total}-I_{\beta }}\times 100\;$$

where $X_{c}$ is the crystallinity index, $I_{PCL}$ is the sum of the intensity of the two main peaks related to PCL, $I_{\beta }$ is the cumulative intensity of the peaks related to β-carotene, and $I_{total}$ is the total integrated intensity.

#### 2.2.6. Differential Scanning Calorimetry

Differential Scanning Calorimetry (DSC) was performed using a DSC 2920 (TA Instruments, New Castle, DE, USA) at a heating rate of 10 °C min^−1^ in a nitrogen atmosphere. Spectra were analyzed using commercial software (Origin 8.5, Originlab Corp., Northampton, MA, USA).

#### 2.2.7. Mechanical Testing

The mechanical properties of the different scaffolds were measured using a tensile tester (STA-1150, ORIENTEC, Tokyo, Japan). The samples, specifically prepared for mechanical testing, featured two reinforced clamping regions on both sides and had an initial testing length of 20 mm. The specimens were then stretched at a tensile rate of 10 mm/min, at room temperature. The tensile strength, elongation at break, and tensile modulus were given by the tensile tester after obtaining the stress–strain curve, while the toughness was calculated using commercial software (Origin 8.5, Originlab Corp., Northampton, MA, USA). The measurements were repeated six times for each group of samples.

### 2.3. Biological Testing

#### 2.3.1. Cell Culture

The BJ-1 cell line was subcultured maintaining a seeding density of 3 × 10^3^ cells·cm^−2^ in DMEM (Minimum Essential Medium Eagle-alpha modification, Gibco, Thermo Fisher, Zürich, Switzerland) with 10% FBS (fetal bovine serum, Gibco, Thermo Fisher, Zürich, Switzerland), 100 U·ml^−1^ penicillin, and 100 μg·ml^−1^ streptomycin (Gibco). The medium was changed every second day. BJ-1 cells were maintained at 37 °C in a 5% CO_2_ humidified atmosphere. This culture was used to address the biocompatibility of β-carotene/PCL composites before electrospinning.

KUSA-A1 cells (JCRB, Osaka, Japan) were first cultured and incubated in a medium consisting of 4.5 g/L of glucose DMEM (D-glucose, L-glutamine, phenol red, and sodium pyruvate, Nacalai tesque, Kyoto, Japan) supplemented with 10% fetal bovine serum. The various samples were previously sterilized upon exposure to ethanol and put in a 24-well plate one by one. The cultured cells were then deposited on the samples in the well at a seeding concentration of 10^5^ cells/well. Cells were concentrated into 50 mL of solution and gently deposited on the samples, then incubated for an hour. Then, 1 mL of culture medium was added to each well. An osteogenic medium was used after 24 h. The medium consisted of Dulbecco’s modified Eagle medium (DMEM) supplemented with nominal amounts of the following constituents: 50 mg/mL ascorbic acid, 10 mM b-glycerol phosphate, 100 mM hydrocortisone, and 10% fetal bovine serum. All samples were incubated at 37 °C for up to 10 days. The medium was changed a total of three times.

#### 2.3.2. Cell Testing

BJ-1 cells were tested on PCL-modified material. In order to avoid the adhesion of cells on the tissue culture plate, TC24wells were precoated with 200 µL Agarose 1.5%. After the solidification of agar, PCL+ curcumin samples were located in the wells. Respectively, 250,000 cells in 300 µL were seeded on no-prewet scaffolds (directly on the surface) or on scaffolds that had been prewet for 1 h in medium solution (prewet 1 h). All samples were prepared in triplicates. BJ-1 cells were co-stained with 5 µM Calcein (Sigma-Aldrich, St. Louis, MO, USA, #17783) and 0.625 µg/mL Ethidium Homodimer for 30 min to discriminate between live and dead cells.

For KUSA-A1, the cytotoxicity of the substrates was assessed and compared by analyzing the samples using the standard MTT (3-(4,5-dimethylthiazol-2-yl)-2,5-diphenyltetrazolium bromide) assay. This colorimetric assay utilizes MTT, a yellow tetrazolium salt, which is metabolized by metabolically active cells into a purple formazan product. The conversion of MTT to formazan is directly proportional to cell viability. The formazan solutions were then analyzed using microplate readers (EMax, Molecular Devices, Sunnyvale, CA, USA) at an absorbance wavelength of 570 nm. The resulting optical density (OD) values served as an indicator of cell viability and cytotoxicity.

Osteogenic differentiation was assessed after 10 days in the osteogenic medium by staining cultured KUSA-A1 cells with the green osteocalcin stain (Osteocalcin Mouse anti-Human, PE, Clone, Fisher Scientific, Hampton, NH, USA) following the manufacturer’s instructions. Fluorescence microscopy was used to visualize osteocalcin localization and quantify the stained area (with respect to the total area of the image) using image analysis software (Fiji, release 2.9.0 [36]). This approach avoids potential interference from the red color of β-carotene, allowing for a more accurate assessment of bone formation compared to other common protocols, such as ALP activity assays. As β-carotene is also known to emit fluorescence in the green region, the results had to be post-treated with analysis software (Fiji, release 2.9.0 [36]) to remove the weaker background fluorescence. The test was performed at 10×.

#### 2.3.3. Bacteria Culture

*Escherichia coli* (25922 ^®^ATCC™) (simply *E. coli*, henceforth) was cultured at 37 °C at Kyoto Prefectural University of Medicine using brain heart infusion (BHI) agar (Nissui, Tokyo, Japan). Starting from an initial 1.0 × 10^9^ CFU/mL, the concentration was diluted with phosphate-buffered saline (PBS) at physiological pH and ionic strength. Subsequently, 100 μL of the bacterial suspension at a density of 1 × 10^8^ CFU/mL was spread onto a BHI agar plate. The samples were sterilized by UV and pressed into the bacteria on BHI agar for inoculation. 

#### 2.3.4. Bacteria Testing

After 24 and 48 h of incubation, the samples were washed with PBS and bacterial viability was assessed using a colorimetric assay (Microbial Viability Assay Kit-WST, Dojindo, Kumamoto, Japan). This assay employed a colorimetric indicator (WST-8), which produces a water-soluble formazan dye upon reduction in the presence of an electron mediator. The amount of the formazan dye generated is directly proportional to the number of living microorganisms. Solutions were analyzed using microplate readers (EMax, Molecular Devices, Sunnyvale, CA, USA) through optical density at 600 nm (OD_600_). 

### 2.4. Statistical Analysis

To assess the statistical significance of the differences observed among the groups, we employed a one-way analysis of variance (ANOVA). This approach enabled us to determine whether any of the mean values for the measured outcomes varied significantly across the experimental groups. For each test, we utilized a sample size of *n =* 5 specimens, ensuring adequate statistical power for the analysis.

## 3. Results

**Figure 1:**
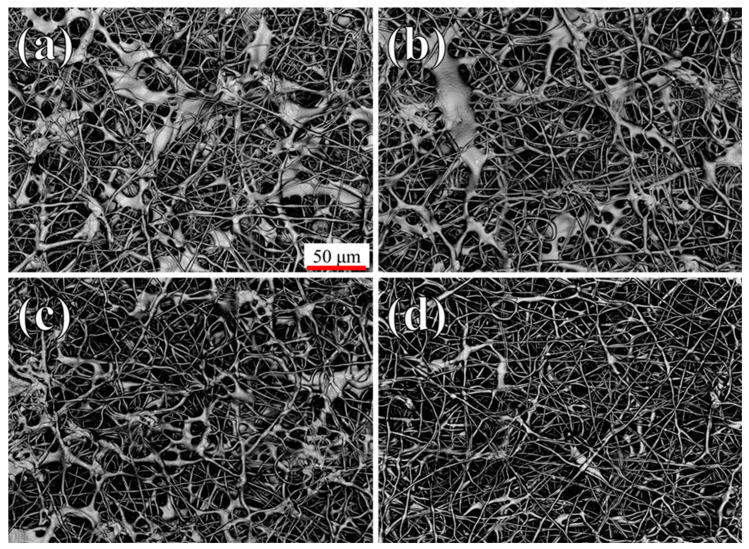
Laser microscope images of the samples at low magnifications: (**a**) pure PCL, (**b**) 1.2% β-carotene, (**c**) 2.4% β-carotene, (**d**) 3.6% β-carotene.

Figure 1 shows the surface morphology of the samples, as obtained at low magnifications using a confocal laser microscope. When the PCL/acetic acid solution is electrospun without any additional additive (Figure 1a), the deposition results in a large amount of elongated droplets, a common defect already observed in a previous work [33], associated with electrospinning that usually indicates an excessive polymer concentration, high viscosity, or a non-optimized electrical field. The quality of the deposition seems to improve progressively with an increase in the concentration of β-carotene, with almost no visible droplets for solutions containing 3.6% (Figure 1d) of β-carotene. 

Fiber fusion at the contact points, another common structural defect for electrospun scaffolds, caused by the evaporation of the solvent, can also be observed on all scaffolds, independently of the presence of β-carotene. 

**Figure 2:**
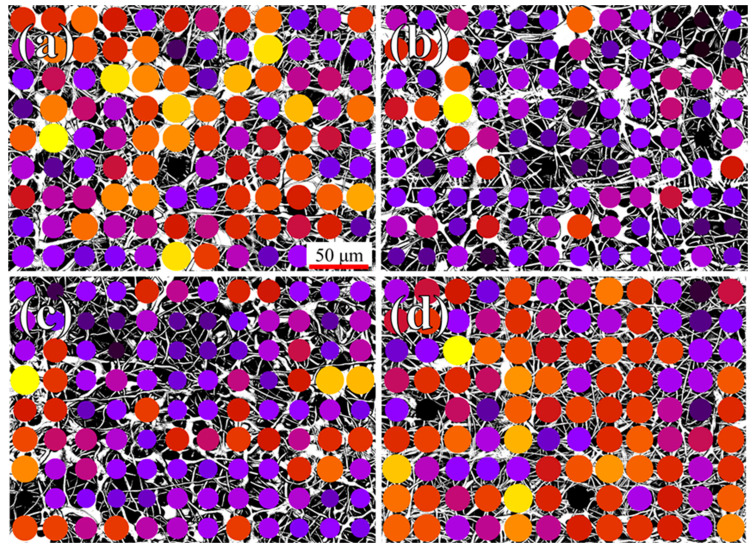
Automatic, software-generated visualization of the fiber density and dispersion based on Figure 1: (**a**) pure PCL, (**b**) 1.2% β-carotene, (**c**) 2.4% β-carotene, (**d**) 3.6% β-carotene.

A qualitative evaluation of the samples’ uniformity is presented in Figure 2. In these four pictures, the images of Figure 1 were transformed into binary black or white, split into 9 x 12 regions, and processed in order to calculate the percentage of white pixels for each region. For visualization purposes, the region with the highest density of electrospun material (higher amount of white pixels) is marked in bright yellow and the lowest in black. From a purely qualitative point of view, the brighter the color of the circle, the higher the material density of the specific sub-region, and the more uniform the colored circles across the surface, the more uniform the density. This simplified representation clearly shows that the lowest average density is achieved with 1.2% of β-carotene, followed by the 2.4% sample. 

**Figure 3:**
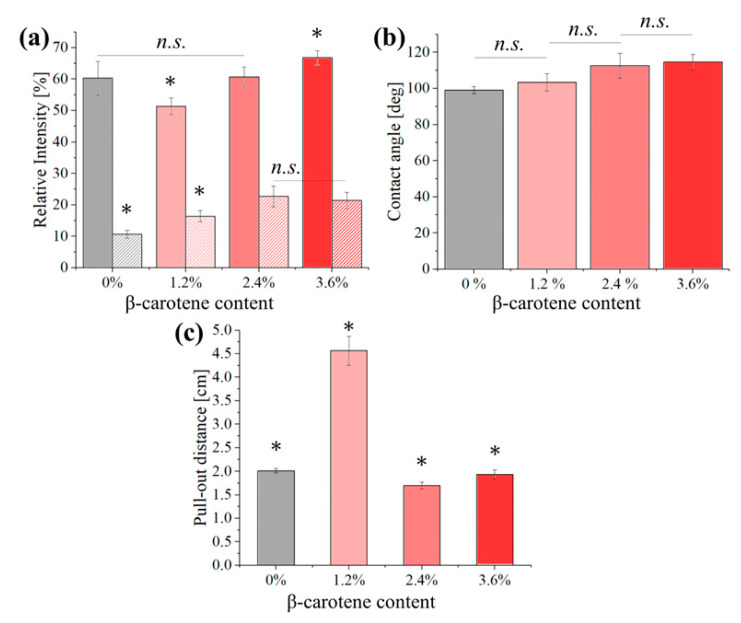
(**a**) Average signal intensity (full) and statistical dispersion of the signal intensity (striped) for the different samples, calculated on the 48 sub-regions of Figure 2. The statistical dispersion of the histograms is based on 5 repetitions on different images. (**b**) Contact angles as measured on the different scaffolds and (**c**) pull-out distance for the different samples. Results marked with “*” have a *p*-value < 0.05 when compared with all others while those marked with “*n.s.*” had *p*-value > 0.05 and were nonsignificant.

A quantification of the fiber dispersion and density observed in Figure 2 is given in Figure 3a, where 100% represents a purely white area (0% of porosity) and 0% a purely black one (no visible fibers), confirming that the sample with 1.2% of β-carotene, which has the darkest circles in Figure 2, has the lowest average fiber density and a low relative dispersion, comparable to that of the PCL reference. From the combined results of Figure 2 and Figure 3a, it seems that the presence of β-carotene progressively increases the density of the scaffolds, while decreasing the amount of droplets.

Figure 3b shows the results of the water contact angle measurements performed on the different samples. PCL is a hydrophobic polymer with a water contact angle usually between 80 and 110°. For the reference scaffold, the value resulted to be about 97° and, despite the large statistical dispersion caused by the inhomogeneity of the scaffold structures, the addition of β-carotene progressively increased the contact angle up to about 112° for a concentration of 3.6%.

Figure 3c shows the results of a pull-out test performed on the four different solutions before electrospinning. This experiment is a semi-quantitative evaluation of the cohesive forces between the molecules of the solution, which would affect both the spinnability and the mechanical properties. It can be observed that the cohesive forces are higher for the composite containing 1.2% β-carotene, followed by pure PCL and then the two composites with higher concentrations of β-carotene. 

**Figure 4:**
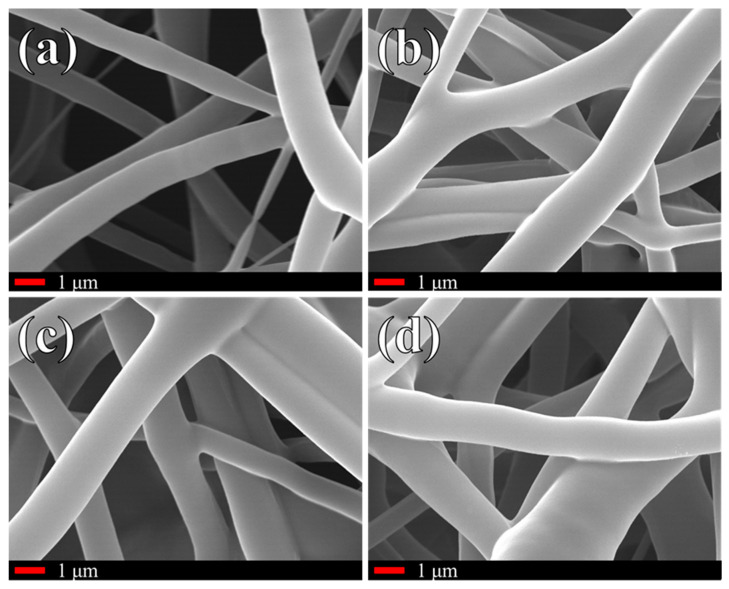
High-magnification micrographs of the electrospun fibers: (**a**) pure PCL, (**b**) 1.2% β-carotene, (**c**) 2.4% β-carotene, (**d**) 3.6% β-carotene.

Figure 4 shows representative images of the four scaffolds at 10 k magnifications. All fibers appear to be smooth, with comparable average diameters. Given the average diameter of β-carotene particles, these should be visible in SEM images, although they cannot be distinguished. The Raman results in the following figures, however, will confirm the incorporation of the powder into the scaffolds. Fiber fusion is clearly visible at all contact points, but the fibers involved retain most of their original geometry. On the microscopic scale, the addition of β-carotene up to 3.6% does not seem to affect the morphology of the scaffold in any measurable way. 

**Figure 5:**
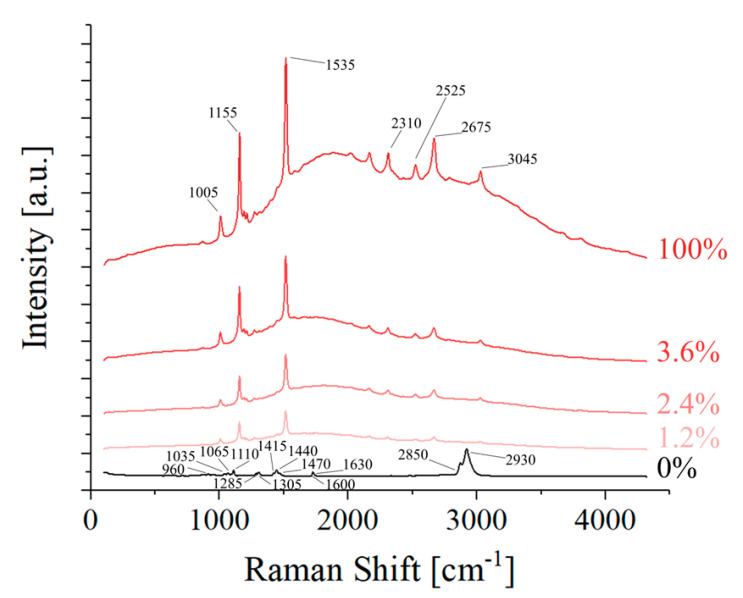
Average Raman spectra of the different samples.

The average Raman spectra acquired on the surface of the scaffolds are presented in Figure 5, as compared to the average spectrum of the pure β-carotene powder. The main bands of these spectra are listed in Table 1 together with their respective assignments. Due to the high Raman cross-section of β-carotene, the bands of PLC are clearly visible only on the reference sample, as the strongest band of β-carotene is about 1000× more intense than the strongest band of PCL, when acquired under the same experimental conditions. In reality, PCL bands are still visible on the three composite scaffolds, but their signal is so low compared to β-carotene that they are easily confused with the background noise. Apart from the main bands of β-carotene, two relatively strong fluorescence bands can also be observed, between 1000 and 4000 cm^−1^. The sum of these bands, centered at about 595 nm and 625 nm, gives the Raman spectrum baseline the same fluorescence emission previously reported in the literature for all-trans-β-carotene [37]. 

Despite the variations in absolute intensity, the relative intensity of the various β-carotene bands observed in Figure 5 is constant across the three composite scaffolds and the reference, meaning that there is no clear evidence of a chemical interaction between the two phases. From the Raman results, it appears that the β-carotene particulate in the scaffolds did not react with PCL and had the same structure as the original powder. The reason why the β-carotene particles cannot be visualized in Figure 4 is probably due to a change in particle size caused by the solvation and subsequent evaporation of the solvent.

**Figure 6:**
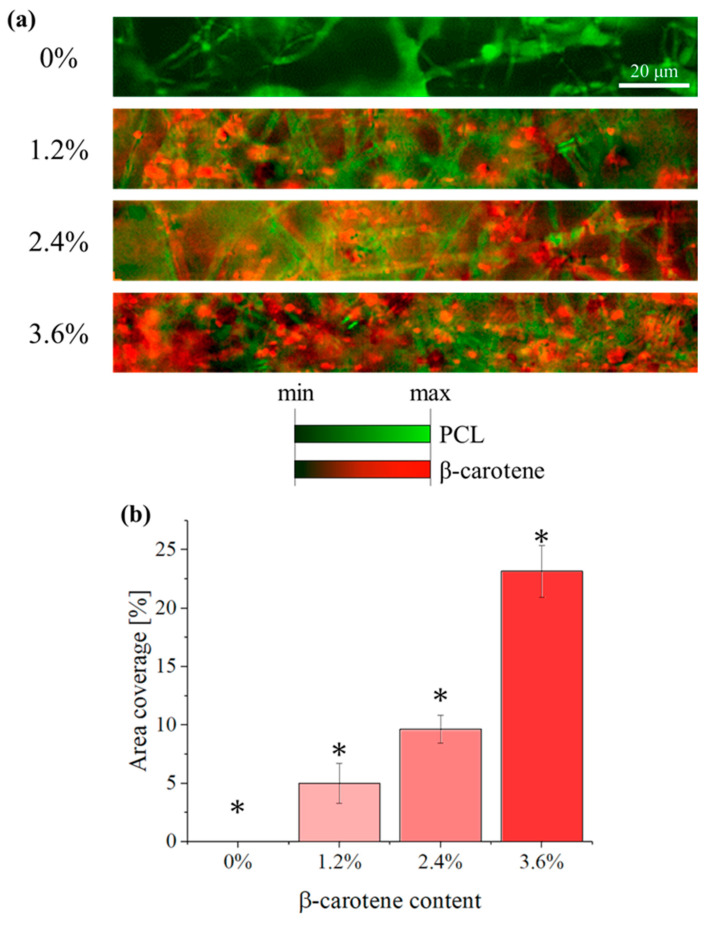
(**a**) Dispersion of β-carotene particulates into the PCL fibers, as observed by using Raman imaging. The β-carotene signal has been filtered with a high-pass filter, so that the signal is at least 100× stronger than that of PCL. (**b**) Area coverage of the β-carotene Raman signal for $I_{1525}\geq 100\;I_{3045}$. Results marked with “*” have a *p*-value < 0.05 when compared with all others.

Despite the relative intensity of the β-carotene signal with respect to PCL, it is still possible to observe signal fluctuations depending on the location. In Figure 6a, portions of the scaffolds are analyzed by Raman imaging, using a high-point-density linear sensor. By applying a high-pass filter to the β-carotene signals, so that the intensity is recorded only if at least 100× stronger than that of PCL, we can observe a dispersion of red dots on the otherwise green PCL polymeric fibers. These red dots, which are not visible in the microscopic images of Figure 1 and Figure 4, represent the locations where β-carotene powder particles are embedded into the PCL matrix. It can be observed that the average intensity and the overall amount of red pixels in Figure 6a are dependent on the concentration of β-carotene, as expected, suggesting that the filtering method used could effectively discriminate between particulates actually present at the investigated location and Raman signals from deeper regions of the sample.

When the amount of β-carotene increases, so does the uniformity of its distribution on the fibers. For the sample containing 1.2% particulates, the red pixels are concentrated in one area, on the left. For a concentration of 2.4%, the red pixels are in most areas, but a few completely green fibers are still visible. When the concentration reaches 3.6%, the distribution results in being relatively uniform across the whole image.

Figure 6b shows the distribution of β-carotene inside the composite, expressed as red pixel area coverage. As the images of Figure 6a were acquired using an arbitrary filtering method, these measurements are to be considered qualitative, but give an effective and immediate way of comparing the three composites. Despite the amount of particulate being increased linearly, the area coverage measured by Raman imaging appears to increase exponentially. This effect can be due to irregularities in the distribution of the particulate inside the polymeric matrix, but also a consequence of the filtering process. 

**Figure 7:**
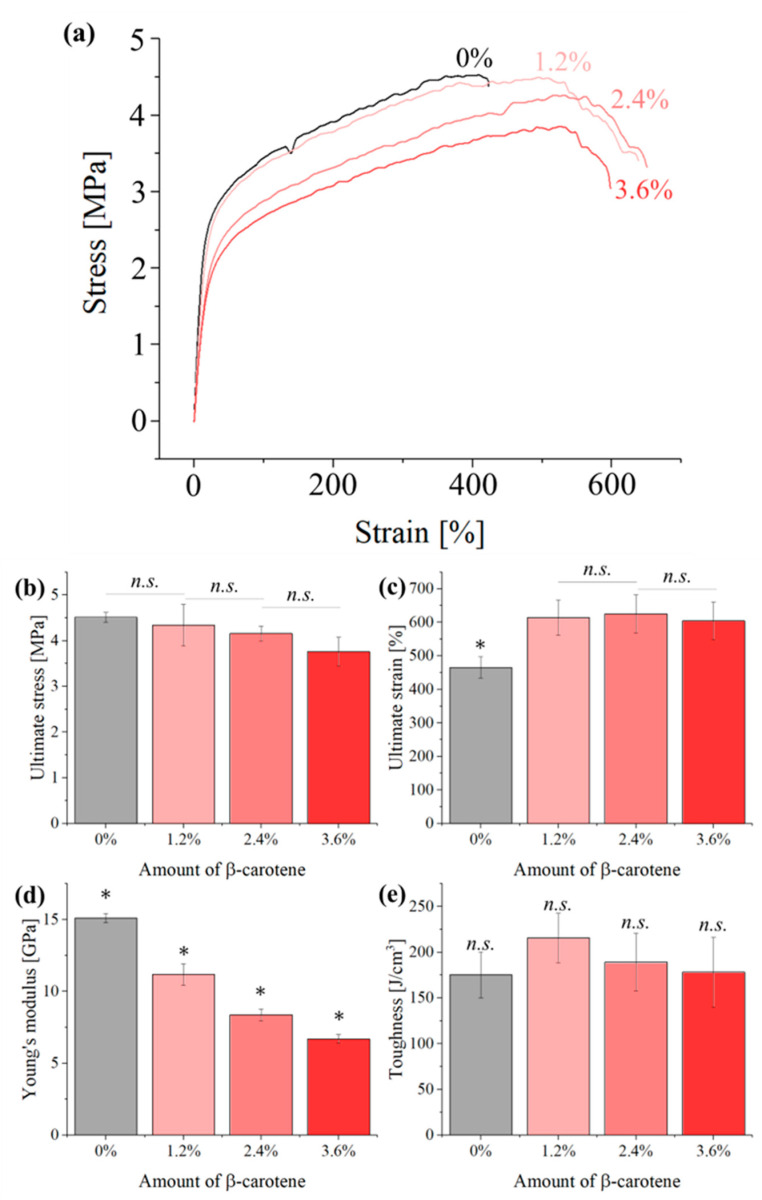
(**a**) Representative stress–strain curves for the different samples, as obtained from tensile testing. Main parameters extrapolated from the tensile testing experiments: (**b**) ultimate strength, (**c**) ultimate strain, (**d**) Young’s modulus, and (**e**) toughness for the different samples. Results marked with “*” have a *p*-value < 0.05 when compared with all others while those marked with “*n.s.*” had *p*-value > 0.05 and were nonsignificant.

Representative stress–strain curves for the four different samples are shown in Figure 7. The addition of β-carotene to the PCL matrix leads to a progressive decrease in ultimate strength, coupled with a slight increase in ultimate strain. These results suggest that β-carotene might act as a plasticizer for the PCL matrix, but its effects are limited and not dose dependent. 

The main parameters associated with the mechanical properties of the scaffolds that can be extrapolated from the stress–strain curves are resumed in Figure 7, in panels from b to e.

As observed in Figure 7, the ultimate stress (Figure 7b) progressively decreases with the amount of β-carotene particulates, while the elongation at break (Figure 7c) initially increases by about 25% but then remains stable up to 3.6% particulates, as the results of the three composites show no statistically significant difference between each other. The Young’s modulus (Figure 7d) of the four materials also decreases, following the same trend previously observed for the ultimate stress, with the composite containing 3.6% of β-carotene showing less than half the modulus of the pristine PCL reference. The increased elongation at break of the composites also results in an increased toughness, but the results appear to be affected by a large statistical dispersion, as the toughness is influenced by the scattering of both ultimate stress and elongation at break.

The decrease in mechanical strength and Young’s modulus suggests that β-carotene weakens the structure of the PCL matrix, possibly due to the interruption of the polymeric chains. This result suggests that the amount of additive should be kept as low as possible to avoid compromising the mechanical properties and stability of the material.

**Figure 8:**
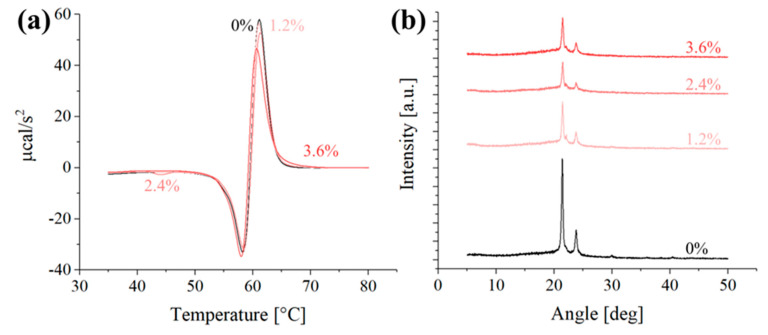
Representative (**a**) first derivative DSC curves and (**b**) XRD diffraction pattern for the different samples.

In the DSC analysis, while the peak melting temperature remains relatively constant across all samples (Figure 8a), a notable decrease in peak intensity is observed with increasing β-carotene content. From pure PCL at ~60 µcal/s^2^, the intensity progressively declines to ~45 µcal/s^2^ in the 3.6% β-carotene composite. One possible explanation for this phenomenon, as previously hypothesized, is that β-carotene acts as a plasticizer and disturbs the crystal formation, increasing the amorphous fraction within the PCL matrix. This increased amorphous content melts at a wider temperature range with lower enthalpy compared to the crystalline portion, contributing to a smaller and broader peak in the DSC curve. 

X-ray diffraction patterns (Figure 8b) further support the hypothesis of β-carotene acting as a plasticizer, as the addition of 1.2% of reinforcement powder causes a drop in the intensity of the peaks related to the (200) and (110) orientations [34]. When more β-carotene is added to the composite, the intensity of the diffraction pattern further decreases (2.4%), before stabilizing (3.6%). These results further support the hypothesis that β-carotene particulate lowers the amount of ordered phase in the PCL polymer.

**Figure 9:**
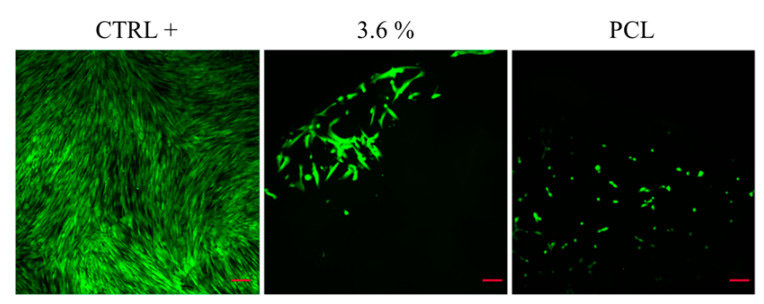
BJ-1 cell line seeded directly on cell culture plates, PCL, or PCL with 3.6% of β-carotene.

**Figure 10:**
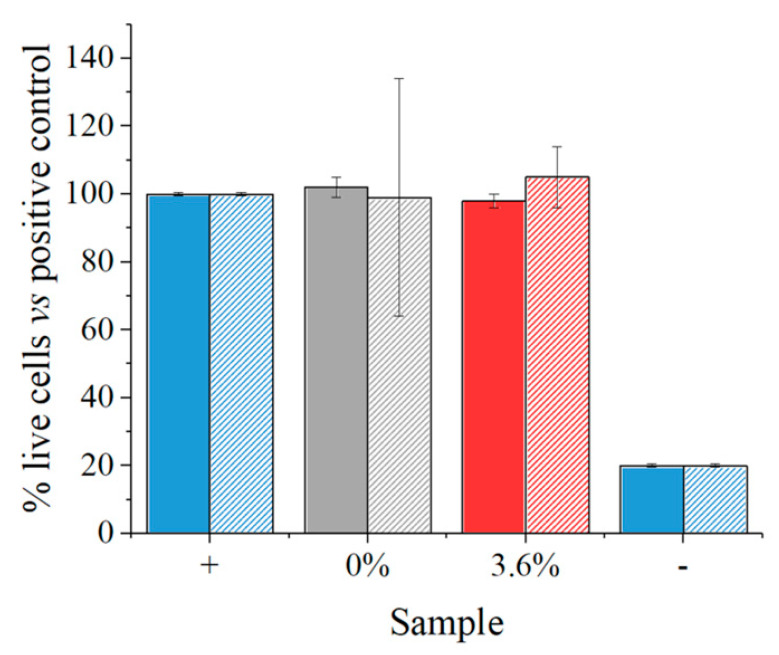
Indirect test of toxicity of material immersed for 24 h in cell culture medium and then transferred to BJ-1 cells. Toxicity was tested after 24 h and 72 h.

The results of the biological testing performed on the four different scaffolds are summarized in Figure 9 and Figure 10. 

The BJ-1 cells commonly used for toxicity assays following ISO 10993-5 [41] guidelines were directly seeded onto PCL or PCL modified with β-carotene. The control used was a cell culture plate, obviously highly efficient for cell culture adhesion and proliferation. Therefore, our gold standard (CTR +) was established based on this control. Three days after seeding BJ-1 cells on the constructs, previously placed in wells coated with 1.5% agar to prevent adhesion to the well surface, live and dead staining was performed using Ethidium Homodimer and Calcein. These markers indicate cell integrity and vitality (Calcein) and cell death (Ethidium Homodimer), respectively, as they bind to the DNA of damaged cells. 

The results presented in Figure 10 highlight that the cells adhered to the plate and showed no distress (CTR+), demonstrating cellular quality as a control. Interestingly, after 3 days, cells on PCL appeared rounded compared to those on PCL modified with β-carotene. From a cellular perspective, this could be explained by the β-carotene modification influencing the hydrophilicity of the material, promoting cell adhesion and subsequent proliferation. Cells on PCL β-carotene, in fact, exhibited a more even distribution and morphologically resembled the CTR, which, of course, showed higher density due to the optimized surface.

However, it was intriguing to observe that, despite not performing a prewetting step, which is typically necessary for PCL due to its hydrophobic nature, the modification with carotene significantly enhanced cell adhesion. Therefore, it is plausible that this modification, by augmenting both adhesion and proliferation, enables direct cellular attachment.

**Figure 11:**
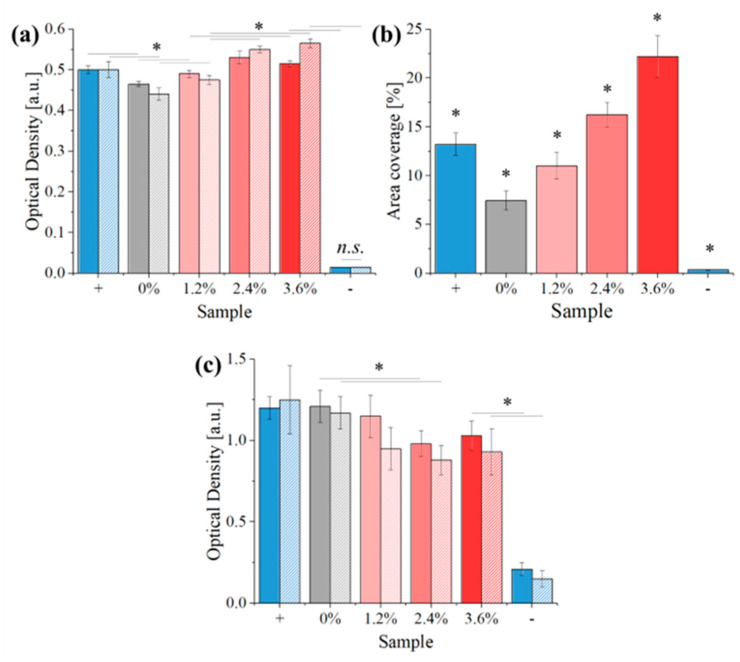
Biological testing results with KUSA-A1 and *E. coli*: (**a**) KUSA-A1 cell optical density, (**b**) area coverage of the osteocalcin staining with respect to the investigated area of the sample, (**c**) *E. coli* optical density. Results marked with “*” have a *p*-value < 0.05 when compared with all others.

As observed in Figure 11a, the amount of KUSA-A1 cells, recorded at both 24 and 72 h of culture, increases with the concentration of β-carotene and is always comparable if not superior to the control (cell culture without samples) for all composite materials. This indicates that the material is not cytotoxic, also suggesting that it potentially stimulates cellular proliferation. The number of cells shows a small decrease going from 24 to 72 h for concentrations of β-carotene equal or inferior to 1.2%, while the opposite is true for higher concentrations.

At 10 days, the scaffolds containing β-carotene are all partially covered by newly formed bone tissue, as indicated by the presence of osteocalcin (Figure 11b). The amount of osteocalcin grows with the concentration of β-carotene, suggesting that the reinforcement also influences bone tissue formation. This can be caused either by an increased cellular adhesion to the substrate or by a direct metabolic effect of the β-carotene. When compared to the positive control (in this case, a disc of titanium grade 5 alloy), the PCL reference shows a decrease in the osteocalcin content, and even an addition of 1.2% β-carotene does not bring the composites on par with the titanium reference. The sample containing 3.6% β-carotene, on the other hand, has almost double the area coverage of the positive control.

When tested with *E. coli* for 24 and 48 h of culture (Figure 11c), the scaffolds show a decrease in optical density (OD) with increasing β-carotene concentration, suggesting reduced bacterial colonization and proliferation. The maximum observed reduction in OD reaches 20% at a concentration of 3.6%. This implies a potential weak antibacterial or bacteriostatic effect associated with the presence of β-carotene within the scaffolds.

## 4. Discussion

Morphological and chemical analyses performed on the electrospun scaffolds indicate that PCL fibers incorporating up to 3.6% of β-carotene can be effectively produced with minimal effort. Still, thermogravimetric analysis and X-ray diffraction indicate that the presence of β-carotene particulate adversely affects the ordered fraction of the PCL structure, in particular at elevated concentrations. 

Despite incorporating β-carotene particulate into the PCL solution prior to electrospinning, significant chemical bonding between these molecules might not have been achieved due to several factors. Firstly, the utilization of acetic acid as the solvent might not have facilitated strong interactions between the highly hydrophobic β-carotene and the semi-crystalline PCL, owing to their differing polarities. Secondly, the relatively short processing time and low temperatures associated with electrospinning might not have provided sufficient energy or activation for potential covalent bond formation, even if suitable functional groups were present. Additionally, the physical form of the β-carotene (powder) could have influenced its dispersion within the PCL matrix, limiting potential contact points for interaction. 

While weaker van der Waals forces or π-π stacking interactions might still be present, exploring alternative solvents or processing techniques in future studies could promote stronger interactions and potentially increase the mechanical properties of the electrospun fibers.

For what concerns the biological response, β-carotene presents a complex interplay between its antioxidant and pro-oxidant behavior, influenced by factors like concentration, cell type, and environmental conditions [42]. While its antioxidant properties might protect cells from harmful reactive oxygen species (ROS), excessive β-carotene can have pro-oxidant effects at higher concentrations or under specific conditions.

Interestingly, in this research, the addition of β-carotene to PCL scaffolds promoted both cellular proliferation and bone formation. Such observations align with previous studies [32,43,44,45], but the topic is still controversial. At moderate concentrations, β-carotene’s antioxidant activity might protect cells from ROS-induced damage, creating a more favorable environment for proliferation. β-carotene has also been reported to enhance the expression of Runx2, ALP, and osteopontin mRNA [46], increase early osteoblastic differentiation, and reduce bone loss [47] by regulating osteoclastogenesis, but higher doses can also result in cytotoxicity, in particular for cells with altered metabolic pathways, an effect that has been extensively studied for the treatment of tumors [48,49]. Further studies have demonstrated that oxidized β-carotene is cytotoxic and inhibits the mitochondrial function in various cell lines [50].

The bioactive effects reported in this research are in line with results previously obtained on fibers containing up to 4% of β-carotene [32], suggesting that these amounts are under the threshold for potential cytotoxicity in human cells. Moreover, it has been demonstrated that β-carotene stimulates mineralization as OCP and TCP are produced near the areas where β-carotene exists. Through the production of HA precursors, HA seed crystals are generated and mature into hard tissues by taking Ca^2+^ and PO_4_^3-^ from the extracellular fluid [51].

For what concerns the antibacterial effect observed in Figure 11c, the reduction is much less pronounced than what was previously observed in the literature [52]. This is partially due to the relatively low concentration of β-carotene inside the scaffolds, but it should be noted that the best results from the literature were observed in carotenoids extracted by plants [53], bacteria [54], and animals [55], which potentially contain other bioactive substances that might play a synergic effect. There is no reason to believe the chemical structure and/or the biological activity of naturally extracted carotenoids to be different from laboratory standards.

## 5. Conclusions

Fibers of PCL functionalized with up to 3.6% β-carotene could be easily electrospun from an acetic acid-based solution. This method yields fibers morphologically comparable with those of the pristine PCL polymer, but with an increased plasticity, a lower ultimate strength, and a decreased elastic modulus.

The loss of mechanical properties may be attributable to the composite structure, in particular a sharp reduction in PCL crystallinity, suggesting that β-carotene disrupts the structure of the polymeric matrix without forming strong bonds.

The composites demonstrate enhanced bioactive properties, notably in cell proliferation and bone tissue formation, along with a limited bacteriostatic effect when tested against *E. coli*.

These findings indicate that PCL composites containing β-carotene hold promise for biomedical applications. Nonetheless, it is crucial to carefully control the β-carotene concentration, as elevated levels may manifest cytotoxic effects. Further research should explore methods to improve the bonding between PCL and β-carotene. This could involve exploring alternative solvents or the incorporation of bonding molecules to create a more cohesive and mechanically robust composite.

## Figures and Tables

**Table 1:** Assignment of the Raman bands presented in Figure 5, with relevant literature references.

Position [cm^−1^]	Vibrations	Origin	References	Position [cm^−1^]
3045	${2v}_{1}$	β-carotene	[38]	3045
2930	$v_{aCH}$	PCL	[39]	2930
2850	$v_{sCH}$	PCL	[39]	2850
2675	$v_{1}+v_{2}$	β-carotene	[38]	2675
2525	$v_{1}+v_{3}$	β-carotene	[38]	2525
2310	${2v}_{2}$	β-carotene	[38]	2310
1525	$v_{1C=C}$	β-carotene	[38]	1525
1470	$\delta_{CH_{2}}$	PCL	[40]	1470
1440	$\delta_{CH_{2}}$	PCL	[40]	1440
1415	$\delta_{CH_{2}}$	PCL	[40]	1415
1305	$\tau_{CH_{2}}$	PCL	[40]	1305
1285	$\tau_{CH_{2}}$	PCL	[40]	1285
1155	$v_{2CC}$	β-carotene	[38]	1155
1110	$v_{CC}$	PCL	[40]	1110
1065	$v_{CC}$	PCL	[40]	1065
1035	$v_{CC}$	PCL	[40]	1035
1005	$v_{3CH}$	β-carotene	[38]	1005
960	$v_{C-COO}$	PCL	[40]	960

## Data Availability

The raw data supporting the conclusions of this article will be made available by the authors on request.
